# Arula-7 powder improves diarrhea and intestinal epithelial tight junction function associated with its regulation of intestinal flora in calves infected with pathogenic *Escherichia coli* O_1_

**DOI:** 10.1186/s40168-023-01616-9

**Published:** 2023-08-05

**Authors:** Hao Chen, Zhifeng Jia, Meiling He, Aorigele Chen, Xin Zhang, Jin Xu, Chunjie Wang

**Affiliations:** 1 College of Animal Science, Inner Mongolia Agricultural University, Hohhot, 010018 People’s Republic of China; 2 College of Veterinary Medicine, Inner Mongolia Agricultural University, Hohhot, 010018 People’s Republic of China; 3 Animal Disease Prevention and Control Center of Bazhou District, Bazhong, China; 4 College of Basic Medical, Inner Mongolia Medical University, Hohhot, 010110 People’s Republic of China; 5 Henan Houyi Bio-Engineering, Inc, He Nan, 451161 Zhengzhou, People’s Republic of China

**Keywords:** Arula-7 powder, Pathogenic *Escherichia coli* O_1_, Diarrhea, Intestinal flora, Intestinal barrier function, Calf

## Abstract

**Background:**

The effects of Arula-7 powder (ASP) on diarrhea and intestinal barrier function associated with its regulation of intestinal microflora in calves infected with pathogenic *Escherichia coli* O_1_ (*E. coli* O_1_) were studied.

**Method:**

Twenty Holstein calves were randomly divided into four treatment groups: normal control (NC), model control (MC), 0.5 mg/kg ciprofloxacin (CIP) and 2.50 g/kg ASP groups.

**Results:**

ASP inhibited the relative abundance of *Proteobacteria*, *Selenomonadales*, and *Enterobacteriales*, and increased the relative abundance of *Lactobacillus*, *Faecalibacterium*, and *Alloprevotella*. Moreover, we demonstrated for the first time that the ASP and CIP promoted weight gain, reduced the diarrhea rate (*P* < 0.05), and enhanced antioxidant capacity (*P* < 0.05) due to the increase in average daily gain (ADG), total protein (TP), and albumin (ALB). In addition, ASP and CIP increased the expression of Zunola occludens-1 (ZO-1), Occludin, and Claudin-1 in the ileum (*P* < 0.05), and improved immunity due to increase levels of interleukin-2 (IL-2), interleukin-4 (IL-4), interferon-γ (IFN-γ), immunoglobulin A (IgA), and immunoglobulin G (IgG) in the serum, strengthened CD4^+^T levels in the ileal mucosa and reducing CD8^+^T and CD11c^+^T (*P* < 0.05).

**Conclusion:**

Hence, The intestinal microbiota environment formed by early intervention of ASP powder has a protective effect on the intestinal mucosal function of calves infected with pathogenic *E. coli*.

Video Abstract

**Supplementary Information:**

The online version contains supplementary material available at 10.1186/s40168-023-01616-9.

## Introduction

Pathogenic *Escherichia coli* (*E. coli*) harms humans and livestock health and causes economic losses to the breeding industry [1]. Pathogenic *E. coli*, the clinical symptom of diarrhea, is very harmful to the health of infants (diarrheal disease leads to 15% of deaths in children under 5 years of age) and newborn poultry, and antibiotics are the first choice for clinical prevention [2]. Currently, many toxicities and side effects (such as destruction of the intestinal flora) caused by the abuse of antibiotics in the livestock industry has led to the refusal of people to consume the foods or products from livestock treated with antibiotics [3]. Therefore, to reduce and prohibit the use of antibiotics, researchers have begun to work on the research and development of alternatives to traditional antibiotics [4]. In this process, researchers have found that herbal medicines have antibacterial, and anti-inflammatory, properties, improve body immunity, and regulate intestinal flora, and herbal medicines maintain the dynamic balance of the internal environment to achieve disease prevention and treatment [5]. It has already been proven that in the process of infection with pathogenic *E. coli* O1, animals suffer from atrophy of villi in the small intestine and diarrhea, which affect the weight gain [6]. Some studies have shown that the two important effects of infection with pathogenic *E. coli* are the change in animal intestinal structure and imbalance of intestinal flora [7]. In fact, pathogenic *E. coli* leads to impaired intestinal epithelial barrier function and induces the destruction of calf gut microbiota balance. Once the microbiota balance is broken, potential pathogens in the intestine invade and colonize the gut, causing various diseases, especially inflammation and diarrhea [8]. Defects in microbial and immune function in the gut of newborn calves, and pathogenic microbes colonize the intestine to increase the risk of diarrhea at this stage [9]. In dairy farming, the pathogenic microbial-induced diarrhea rate reaches 60 ~ 70%, the mortality rate is more than 50%, and sick calves also emit a large amount of pathogenic bacteria to the outside world [10]. In addition to gut maturation, the calf has to adapt to the new environment, which is loaded with pathogenic microbes that may cause the calf to have diarrhea and die [11, 12].

Mongolian medicine plants, including seeds, berries, roots, stems, leaves, bark, and/or flowers from one or more plants, have therapeutic or other human health benefits. In some traditions, the scope of Mongolian medicine has been extended to cover materials of fungal, mineral and animal origin [13]. Herb compound intervention provides a feasible and practical method for mitigating intestinal inflammation and regulating animal intestinal flora [14]. There is an overwhelming consensus based on countless evidence that the herbal compound has the beneficial effects of improving the weight gain, enhancing immunity of livestock, protecting the intestinal mucosal barrier, and reducing disease [15]. Additionally, the favorable effects of curcumin on the animal health include the production of metabolites that inhibit the colonization of pathogenic microorganisms, which regulate the immunity of the animal’s body, alleviate intestinal inflammation, and improve the intestinal barrier [16, 17]. Arula-7 powder (ASP), a traditional Mongolian medicine formula chronicled in the Mongolian medicine division, is widely used to improve intestinal diseases, such as diarrhea. Because ASP has long been used for the prevention and treatment of human diseases, research on its use by veterinarians is in a low-level development state.

Supplementation with short-chain fatty acids produced by specific bacteria significantly increased the production of Treg cells and enhanced the antibacterial activity of macrophages to prevent colitis [18]. Functionally, there is overlap in Treg inhibitory functions, and a subpopulation of T cells co-expressing CD8 and CD11c that represent an important category of adaptive immunomodulators with the ability to inhibit and exert effects in vivo was recently reported [19]. Numerous studies have confirmed that CD4 + and CD8 + T cells accelerate the activation and proliferation of T cells and produce various cytokines [20]. It is generally believed that inflammation plays a key role in interleukin (IL) and immunoglobulin (Ig) production to generate body immunity [21]. In recent years, there have been many studies on CD4 + T lymphocyte subsets and their representative factors in diarrheal diseases, but few studies have been conducted on bacterial diarrhea. Therefore, this study will evaluate the effects of ASP on the status of CD4 + T lymphocytes and their representative factors in mammals.

It has long been known that infection with pathogenic *E. coli* can damage the structure and function of the gut of the gut microbiota of an animal. However, there are few reports on the effect of herbal powder on intestinal epithelial tight junction function and intestinal flora, and the beneficial effect of improvement of diarrhea in calves infected with pathogenic *E. coli* O_1_ has rarely been reported. Therefore, in this study, ASP was used to interfere with the effective colonization of early calf gut microbiota, and the effective colonization of pathogenic microbes in the calf intestine was controlled from the source, thus providing suitable colonization conditions for probiotics in the calf.

The purpose of this study was examine the effects of Arula-7 powder on the diarrhea, immunity, intestinal epithelial tight junction function and intestinal flora of calves infected with pathogenic *E. coli* O_1_, thus providing a theoretical basis and experimental evidence for understanding the biological effects of Arula-7 powder and its potential for regulating intestinal flora.

## Materials and methods

### Setting of groups and drug dose

All experiment animals were approved and performed in accordance with the guidelines of Institutional Animal Care and Use Committee of Inner Mongolia Agricultural University (IMAUG00001110). This study used Holstein bull calves (*n* = 8) and female calves (*n* = 12) reared at HeTan Crassland and Cattle 301 Ranch of the Shanxi, China.

The calves used in this study received 4 L of whole milk every day from the first day to the seventh day of the trial. The calves used in the study had no records of respiratory or enteric diseases or use of treatments for any disease. Twenty healthy Holstein calves were randomly divided into the natural control group (NC; bull calves = 2; female calves = 3); model group (MC; bull calves = 2; female calves = 3); Ciprofloxacin (CIP; bull calves = 2, female calves = 3; CIP) and Arula-7 powder group (ASP; bull calves = 2; female calves = 3). Calves in the CIP and ASP groups received the following treatment twice per day for 4 days: 0.5 mg/kg Ciprofloxacin and compound Mongolian medicine 2.50 g/kg Arula-7 powder at a dosage. The NC group did not receive *E. coli* O1 or medicinal treatment, while the calves in all other groups received oral *E. coli* O1 (2.50 × 10^11^ CFU/mL, 100 mL) on day 5, the *E. coli* O1 strain was provided by the Cow Production Laboratory, College of Animal Science, Inner Mongolia Agriculture University. And the calves in the CIP and ASP groups received the medicine treatment until day 7. At the end of the experiment, the calves were humanely euthanized using a captive bolt gun stunning method and sampling was completed within 30 min after euthanasia. According to the color and texture of the calf's feces, a score of 3 or more is determined as diarrhea based on the fecal scoring standard [22].

### Preparation of ASP

All seven medical plants were purchased from Hohhot Tianli Co., Ltd. (Hohhot, China); the detailed composition of ASP is listed in Table 1. The ASP was crushed, and then the powder was filtered through 200 mesh gauze.

**Table 1:** The composition of Arula-7 powder

Chinese name	Latin name	Ratio
He Zi	*Terminalia chebula Retz*	3
Qian Cao	*Rubia wallichiana Decne*	2
Cao Wu Ye	*Folium aconiti kusnezoffii*	2
Gan Song	*Nardostachys chinensis Batal*	2
Ru Xiang	*Boswellia carteri*	2
Qu Mai	*Dianthus superbus L*	1
Fan Bai Cao	*Potentilla discolor Bge*	2

### *DNA isolation and 16S rRNA IonS5*^*TM*^*XL sequencing*

For microbiota analysis, fresh cecal and colon contents (*n* = 5) were collected at days 7 and RNA extraction (1.0 ng/mL) was performed. The extracted total RNA was amplified using CTAB/SDS method and sequencing (the RNA was stored at − 78.5 °C in dry ice and performed at the Novogene Bioinformatics Technology Co., Ltd. Briefly). The 16S rRNA V34 341F 5′-CCTAYGGGRBGCASCAG-3′ and 805R 5′-GGACTACNNGGGTATCTAAT-3′ amplicon was amplified by PCR and sequenced in the IonS5^TM^XL platform using the 2 × 250 bp paired-end protocol [23]. PCR products were purified using the QIA quick Gel Extraction Kit (QIAGEN, Dusseldorf, Germany) [24]. Single-end was used to evaluate the library quality and subsequently sequenced on an IonS5TMXL platform [25]. The 97% similarity sequence was analyzed by Alpha diversity including rarefaction analysis [26]. In brief, the OTU were influenced by IonS5TMXL [27] for the following analysis: alpha diversity, including observed species and Chao1 indices, number of observed OTUs and Shannon and Simpson diversity based on unweighted UniFrac distance of IonS5TMXL sequencing depth [28].

### Analysis of intestinal morphology

After a 7-day trial, all the jejunal and ileal segments of calves were stored at 10% formalin (Bkmam Biotechnology) and embedded in paraffin (Biosharp, BL957A) for histological and immunofluorescence analysis [29]. The paraffin-embedded jejunal and ileal tissue samples were sliced, stained by hematoxylin and eosin (H&E) methods, and then observed under an optical microscope.

### Biochemical analysis

After a 7-day trial, 30 min before euthanasia, Blood samples collected from the venous vein were centrifuged at 3000 × *g* for 15 min at 4 °C, and the clear supernatant was collected, dispensed into 1.5 mL centrifugal tubes and stored at − 80 °C for biochemical analysis. The enzyme-linked immunosorbent assay (ELISA, Bethesda, MD, USA) was used to quantitatively detect the level of SIgA, IgG, IgA, IFN-γ, TNF-α, IL-6, IL-2, and IL-4 in calf serum. Strictly follow the instructions of the kit for each step, and conduct a preliminary study for each experiment.

### Analysis of serum biochemical indices

After a 7-day trial, the levels of serum urea nitrogen (BUN), serum alanine aminotransferase (ALT); aspartate aminotransferase (AST), alkaline phosphatase (ALP), total protein (TP), albumin (ALB), glucose (GLU), and total cholesterol (TC) were analyzed by BS-180VET (Mindray BS-2600 Vet, China), according to the manufacturer’s instructions (Bethesda, MD, USA).

### Intestinal cytokines determined by ELISA

After a 7-day trial, after grinding in liquid nitrogen, total protein was extracted from ileal mucosa samples with lysis buffer (BaoXin, Hohhot, China), and then clarified by centrifugation (3000 × *g* for 15 min at 4 °C). The levels of CD4^+^T, CD8^+^T, CD11c^+^, ZO-1, Occludin, and Claudin-1 were measured using ELISA kit (Bethesda, MD, USA).

### Immunofluorescence and confocal microscopy

At the end of the experiment, the calf jugular vein was bled to death and the ileal was dissected for immunofluorescence experiments. The paraffin sections of the ileal were baked in an oven at 60 °C for 1 h, and then deodorized with xylene I, xylene II and xylene III for 5 min after soaking in 100%, 95%, and 80% ethanol for 5 min. The slides were incubated in biotin-conjugated primary antibody CD4^+^T (1:800 dilution, GeneTex), CD8^+^T (1:300 dilution, Bioss), and CD11c^+^T (1:500 dilution, Bioss), Occludin (1:500 dilution, Proteintech), ZO-1 (1:400 dilution, Proteintech) and Claudin-1 (1:300 dilution, Proteintech) overnight at 4 °C, and secondary antibody (1:200 dilution, Donkey anti Rat 594, Jackson) at room temperature for 1 h. The following steps are described by Xiong and Jin et al. [30, 31]. Finally, fluorescence was visualized using Olympus CK40 laser scanning confocal microscope.

### Statistical analysis

All the data from experiment were analyzed by using the one-way ANOVA. All the results obtained from replicate experiments are showed as means ± s.e.m where “*n*” represents the number of animals/samples, and other figure results are presented with means ± SEM, and histogram using GraphPad Software (La Jolla, CA, USA, www.graphpad.com) [32]. Significance was set at *P* < 0.05.

## Results

### Effects of treatments with ASP and CIP on the intestinal microbiome alpha diversity

Table 2 shows the results of the Chao1 index and Shannon index in the contents of cecum and colon. Compared with MC group, Chao1 index of the cecal contents samples in the 2.50 g/kg ASP were significantly higher (*P* < 0.05). Compared with NC group, the Chao1 index of the cecal and colonic contents samples in MC group decreased apparently (*P* < 0.05), and the Shannon index of the cecal contents in MC group decreased apparently (*P* < 0.05). Compared with the NC group, the Shannon index of the of the cecal contents samples in 0.5 mg/kg CIP group increased apparently (*P* < 0.05). Chao1 index of the samples in the 0.5 mg/kg CIP group was significantly higher than those in the MC group (*P* < 0.05). Compared with NC and CIP groups, Chao1 index of the rectal feces samples in the ASP group were significantly higher (*P* < 0.05). Compared with the MC group, the Shannon index of the of the rectal feces in ASP group increased apparently (*P* < 0.05).

**Table 2:** Effect of ASP on intestinal flora alpha diversity in diarrhea calves

Items	NC	MC	0.50 mg/kg CIP	2.50 g/kg ASP
Cecal content				
Chao1	172.35^a^	128.42^c^	138.05^bc^	160.30^ab^
Shannon	4.00^a^	3.29^c^	3.61^b^	2.54^d^
Colonic content				
Chao1	223.34^a^	141.61^b^	193.50^a^	132.30^b^
Shannon	3.67^a^	3.40^a^	3.87^a^	3.47^a^
Rectal feces				
Chao1	136.18^bc^	211.61^ab^	115.63^c^	231.52^a^
Shannon	3.71^ab^	2.83^b^	2.84^b^	4.0^a^

^a,b,c^Means bearing different superscripts in the same row differ significantly (*P* < 0.05)

### *Overall structure regulation of intestinal flora of calf diarrhea caused by E. coli O*_*1*_* after ASP treatment*

**Fig. 1:**
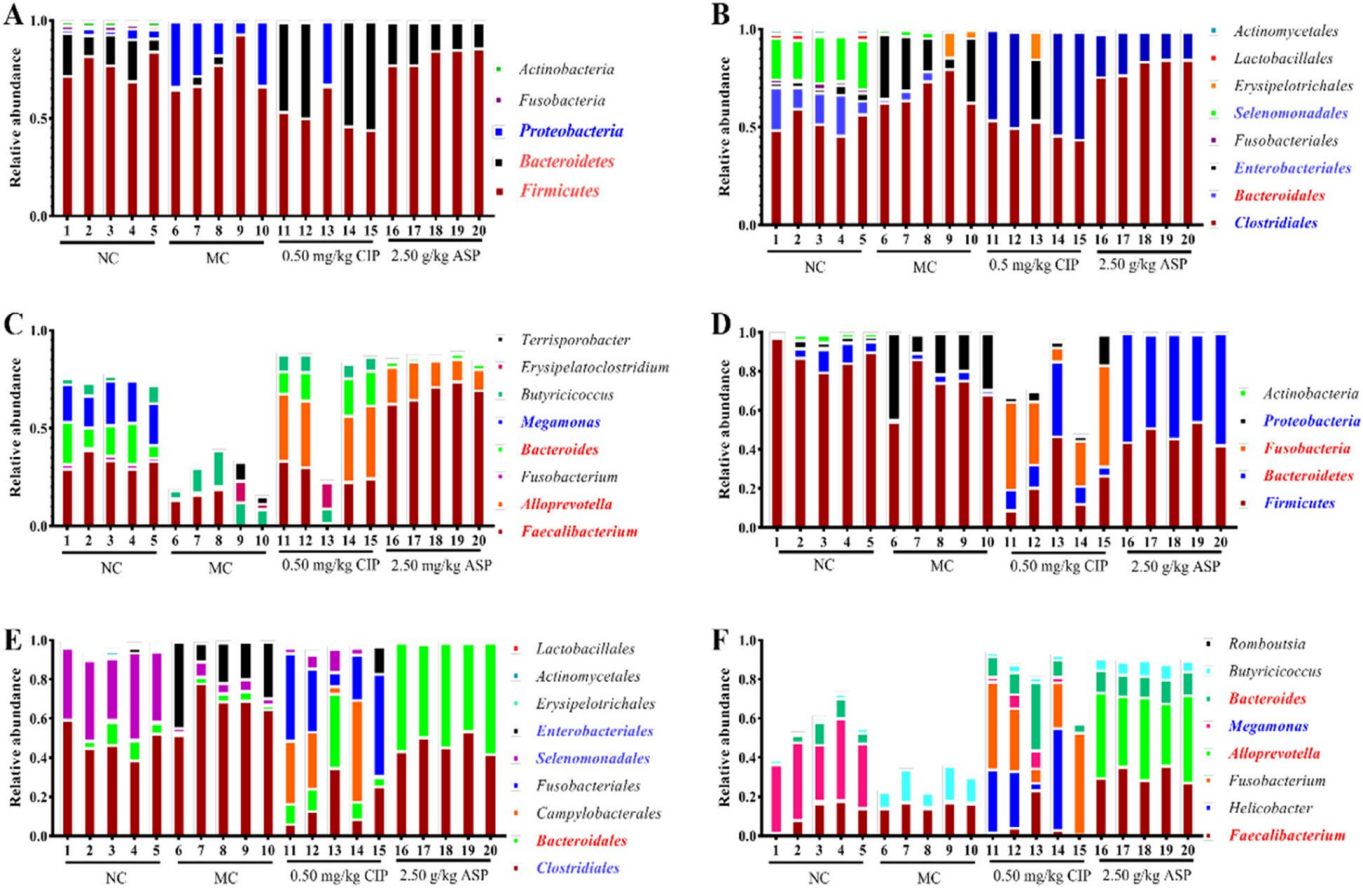
Effect of ASP on the composition of cecal and colonic contents microbiota in calves infected with pathogenic *E. coli* O_1_. Graph** A** ~ **C** represents the OTUs at different taxonomical levels in the contents of the cecal: phylum, Order, and Genus. Graph** D** ~ **F** represents the OTUs at different taxonomical levels in the contents of the colonic: phylum, Order, and Genus. Red Latin indicates a significant increase (*P* < 0.05), and blue indicates a significant decrease (*P* < 0.05)

Histograms illustrating the relative abundances of the three different taxonomic levels of calf cecum and colon contents is shown in Fig. 1 (Phylum, Order, and genus). With the different treatments, we observed a profound change in intestinal flora at the phylum, order and genus levels. In the cecal contents, compared with MC group, 2.50 g/kg ASP increased the relative abundance of *Firmicutes* and *Bacteroidetes*, and decreased *Proteobacteria* in phylum, but 0.50 mg/kg CIP increased the relative abundance of *Bacteroidetes*, and decreased *Firmicutes* and *Proteobacteria* (Fig. 1A). Compared with MC group, the relative abundance of *Clostridiales* and *Bacteroidales* was increased in the ASP group (*P* < 0.05). The relative abundance of *Faecalibacterium*, *Alloprevotella* and *Bacteroides* was significantly different among the ASP and CIP groups (*P* < 0.05). Compared with NC group, the *Proteobacteria* and *Clostridiales* abundance of calf in cecal content was the higher in MC group at day 7.

The relative abundance of *Firmicutes* and *Proteobacteria* decreased in ASP and CIP groups at days 7 in colonic content if compared with MC group (Fig. 1D, *P* < 0.05). At the order level, The relative abundance of *Clostridiales* increased in MC group at days 7 in colonic content if compared with NC group. Compared with MC group, ASP and CIP groups decreased the relative abundance of *Clostridiales* and *Enterobacteriales*, but increased *Bacteroidales* (Fig. 1E, *P* < 0.05).

### *Arula-7 powder alters the intestinal flora of calves infected with pathogenic E. coli O*_*1*_

**Fig. 2:**
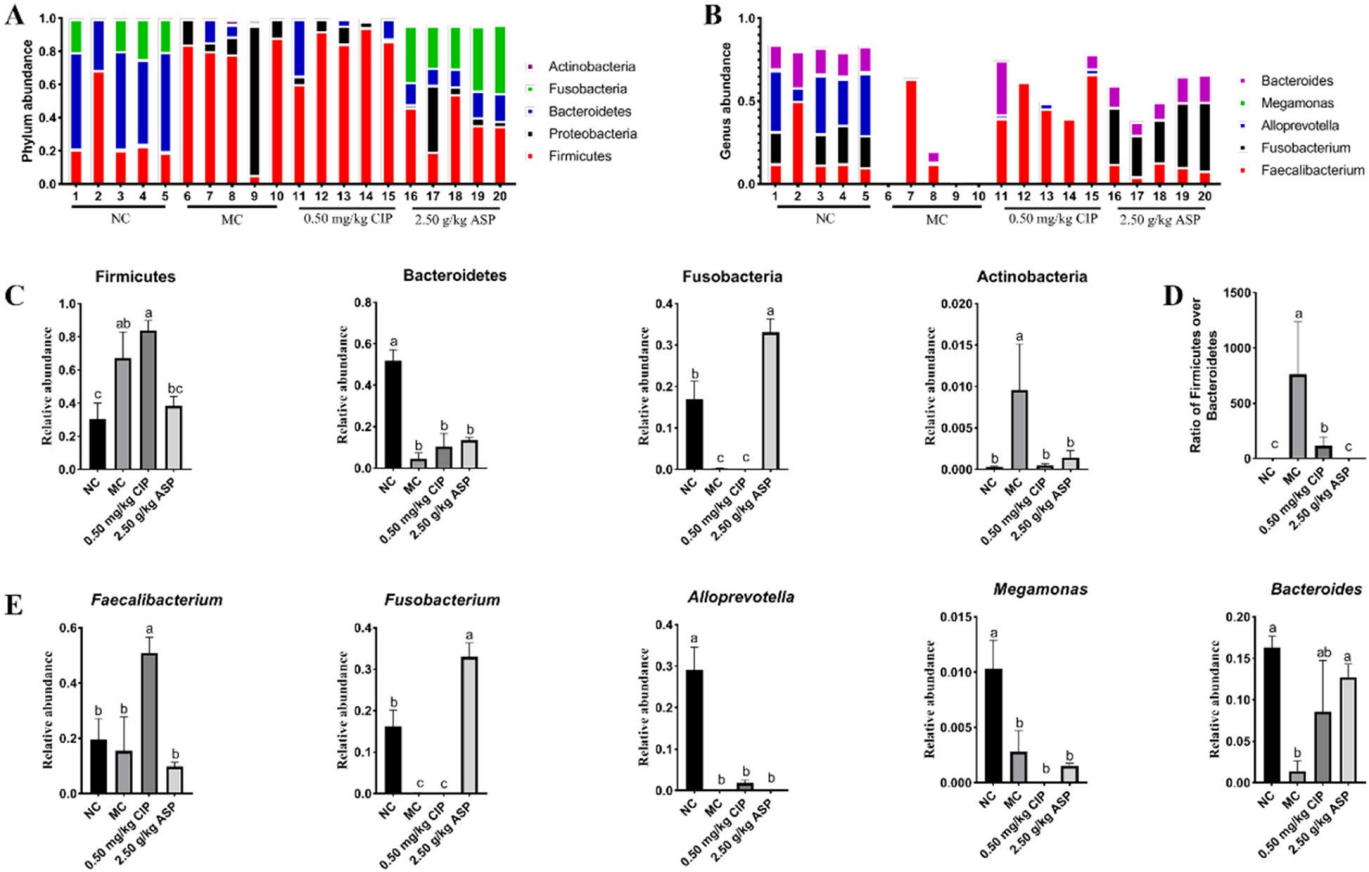
Effect of Arula-7 powder on the composition of rectal microbiota in calves infected with pathogenic *E. coli* O_1_. **A** Graph represents the OTUs at different taxonomical levels: phylum (*n* = 5). **B** Graph represents the OTUs at different taxonomical levels: order (*n* = 5). **C** The change in the relative abundance of phylum Firmicutes, Bacteroidetes, Fusobacteria, and Actinobacteria. **D** The ratio of Firmicutes/Bacteroidetes was significantly low in 2.50 g/kg ASP group. **E** The change in the relative abundance of genus *Faecalibacterium*, *Fusobacterium*, *Alloprevotella*, *Megamonas*, and *Bacteroides*. Metastats analysis was applied to identify the significantly differentially abundant phylum and genera among groups. Different letters above the bars denotes significantly differentially abundant genera among groups. (*P* < 0.05). *n* = 5

To assess how ASP administration affected the calf rectal gut microbial composition, the phylum and genus-level bacterial relative abundance of the control and treatment groups at days 7 was compared (Fig. 2). At the phylum level, compared with MC group, the Firmicutes, Actinobacteria, and ratio of Firmicutes/Bacteroidetes was reduced in ASP group from day 7 after ceased ASP application, and the significantly higher abundance in Firmicutes, Actinobacteria, and ratio of Firmicutes/Bacteroidetes (*P* < 0.05) in MC group was detected, and the markedly more *Faecalibacterium* (*P* < 0.01) was detected after ceased CIP application. At day 7, there is significantly higher *Fusobacterium* and *Bacteroides* (*P* < 0.05).

### Effects of different doses of Arula-7 powder on the content of E. coli in the intestine of calves

We quantified these two important bacterial species in the cecal, colonic, and rectal contents digesta by using quantitative real**-**time PCR analysis. The results are shown in Table 3. The ASP, CIP, and NC groups had a similar relative abundance of *E. coli* in cecal and rectal contents. Moreover, the number of *E. coli* in colonic contents from the ASP and MC groups was significantly higher while the number of *E. coli* was significantly lower than those in the other groups (*P* < 0.05), and the number of *E. coli* in the MC group was significantly higher than those in the other groups (*P* < 0.05).

**Table 3:** Quantitative real-time PCR analysis of total *Escherichia coli* in cecal, colonic, and rectal contents [lgCFU/g wet weight)]

Items	NC	MC	0.5 mg/kg CIP	2.50 g/kg ASP
Cecal content	5.73^c^	12.46^a^	7.06^c^	8.48^b^
Colonic content	6.44^b^	12.47^a^	6.80^b^	10.98^a^
Rectal feces	5.24^c^	9.67^a^	5.42^bc^	6.82^b^

^a,b,c^Means bearing different superscripts in the same row differ significantly (*P* < 0.05)

### Effects of ASP on weight gain, diarrhea rate, and blood biochemical indexes of calves with diarrhea

After a 7-day trial, the growth performance and diarrhea rate of calves with *E. coli* diarrhea are shown in Table 4. Compared with the NC group, MC group significantly decreased final weight (*P* < 0.05) and decreased average daily gain (ADG, *P* < 0.05), but ASP increased ADG (*P* < 0.05), CIP group had no effect on ADG (*P* > 0.05). ASP and CIP groups significantly decreased the diarrhea rate compared with that in MC group (*P* < 0.05). In addition, the ASP and CIP groups restored the calf body weight to the NC group level.

**Table 4:** Effects of ASP on weight gain and diarrhea rate of calves

Items	NC	MC	0.5 mg/kg CIP	2.50 g/kg ASP
Initial weight, kg	29.75 ± 0.73^a^	29.02 ± 1.04^a^	30.85 ± 1.24^a^	29.78 ± 1.04^a^
Final weight, kg	33.37 ± 0.83^a^	31.90 ± 1.25^b^	33.89 ± 0.86^a^	34.15 ± 1.37^a^
ADG, kg/day	0.56 ± 0.08^b^	0.25 ± 0.04^c^	0.46 ± 0.11^b^	0.71 ± 0.05^a^
Diarrhea ratio, %	0.00^c^	80.00^a^	20.00^b^	20.00^b^

^a,b,c^Means bearing different superscripts in the same row differ significantly (*P* < 0.05)

### Effect of ASP on blood biochemical indexes of calves with Escherichia coli diarrhea

**Fig. 3:**
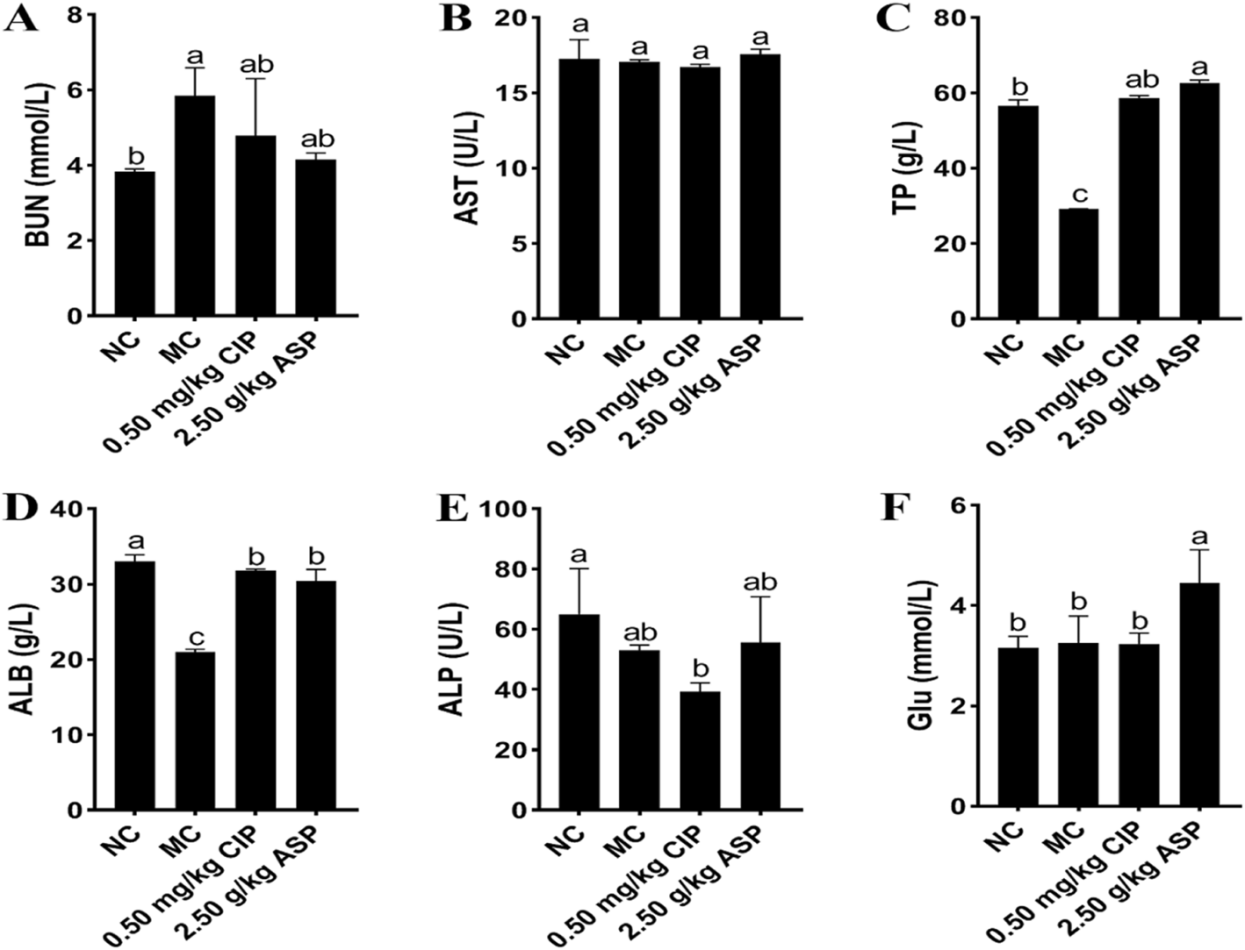
Effect of ASP on blood biochemical indexes of calves with *E. coli* diarrhea. **A** BUN, blood urea nitrogen. **B** AST, aspartate aminotransferase. **C** TP, serum total protein. **D** ALB, albumin. **E** ALP, alkaline phosphatase. **F** Glu, glutamic. *n* = 5. In the same histogram, the values with the same letter superscripts mean no significant difference (*P* > 0.05), while with different letter superscripts mean significant difference (*P* < 0.05)

The blood indexes of calves with *E. coli* diarrhea are shown in Fig. 3. After a 7**-**day trial, compared with the NC group, MC group had no effect on Glu (Fig. 3F) and AST (Fig. 3B) in serum. Moreover, compared with the MC group, ASP, and CIP groups significantly increased TP (Fig. 3C) and ALB (Fig. 3D) and CIP decreased the ALP (Fig. 3E) content (*P* < 0.05), and the level of TP and Glu in ASP group were significantly higher than NC group.

### Effect of ASP on the intestinal morphology of calves with Escherichia coli diarrhea

**Fig. 4:**
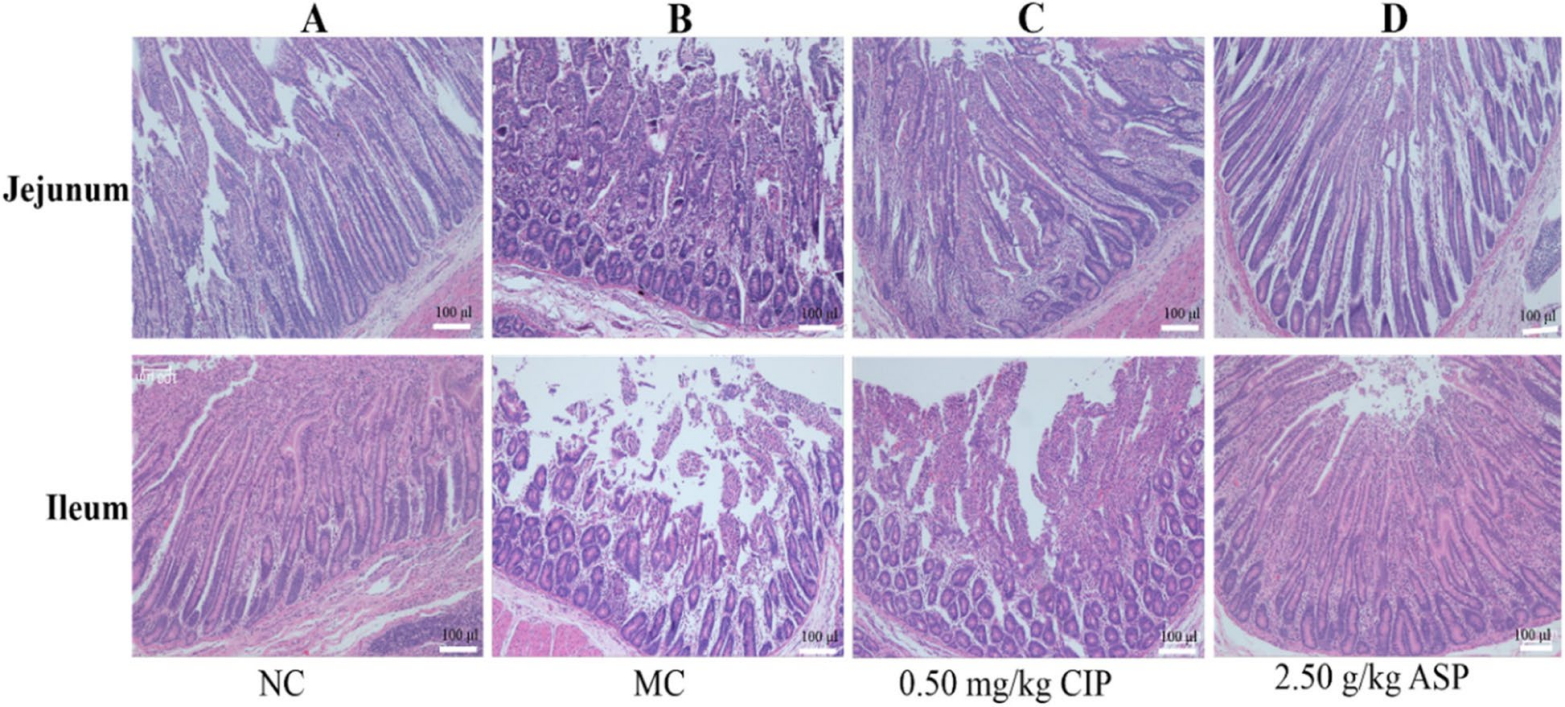
Effects of ASP on histopathological changes of jejunum and ileum with H&E staining (original magnification of 100 ×). **A** Stroke-physiological saline solution + NC group, **B** *E. coli* O_1 _+ MC group, **C** *E. coli* O_1 _+ 0.5 mg/kg CIP group, **D** *E. coli* O_1_ + 2.50 g/kg ASP group

As shown in Fig. 4, compared with the NC group, the intestinal villi in the jejunum and ileum of the MC group with *E. coli* O_1_ were severely shed, especially the ileum injury was the most serious. while the villi in the ASP group were intact and tight.

### Effect of ASP on tight junction protein and lymphocyte factor in the ileum of calves with Escherichia coli diarrhea

To investigate the effects of ASP treatment on calf tight junction proteins and lymphocytes, the ileal ZO-1, Occludin, and Claudin-1, as well as ileal CD4^+^T, CD8^+^T, and CD11c^+^T, were monitored (Table 5). Compared with the NC and MC groups, the level of ileal ZO-1, Occludin, and Claudin-1 were enhanced in ASP group at day 7 (*P* < 0.05). Compared with the NC group, pathogenic *E. coli* O_1_ did result in a significant reduction in the ileal CD4^+^T levels for MC group. However, the level of ileal CD4^+^T was increased in ASP and CIP groups (*P* < 0.05). Compared with MC group, CIP and ASP group significantly reduced the content of CD8^+^T and CD11c^+^T (*P* < 0.05), and the levels of CD4^+^T, CD8^+^T, and CD11c^+^T in the ASP and CIP groups were restored to the NC group level.

**Table 5:** Effect of ASP on tight junction protein and lymphocyte factor in calf ileum

Items	NC	MC	0.5 mg/kg CIP	2.50 g/kg ASP
ZO-1, ng/0.2 g	130.36 ± 6.50^b^	110.0 ± 6.83^c^	133.81 ± 10.42^b^	156.90 ± 11.97^a^
Occludin, ng/0.2 g	707.23 ± 16.37^b^	636.56 ± 20.91^b^	650.98 ± 30.90^b^	1062.52 ± 97.94^a^
Claudin-1, pg/0.2 g	220.28 ± 2.03^b^	214.32 ± 5.28^b^	211.51 ± 6.34^b^	232.04 ± 3.17^a^
CD4^+^, ng/0.2 g	72.39 ± 4.35^a^	59.03 ± 3.80^b^	70.32 ± 1.04^a^	73.39 ± 4.80^a^
CD8^+^, ng/0.2 g	28.50 ± 2.84^ab^	32.19 ± 2.67^a^	25.75 ± 1.21^b^	25.20 ± 3.11^b^
CD11c^+^, ng/0.2 g	45.42 ± 0.54^ab^	43.61 ± 1.79^ab^	47.50 ± 1.17^a^	37.31 ± 0.21^b^

^a,b,c^Means bearing different superscripts in the same row differ significantly (*P* < 0.05)

### Effects of ASP on the expression of intestinal mucosa tight junction protein

**Fig. 5:**
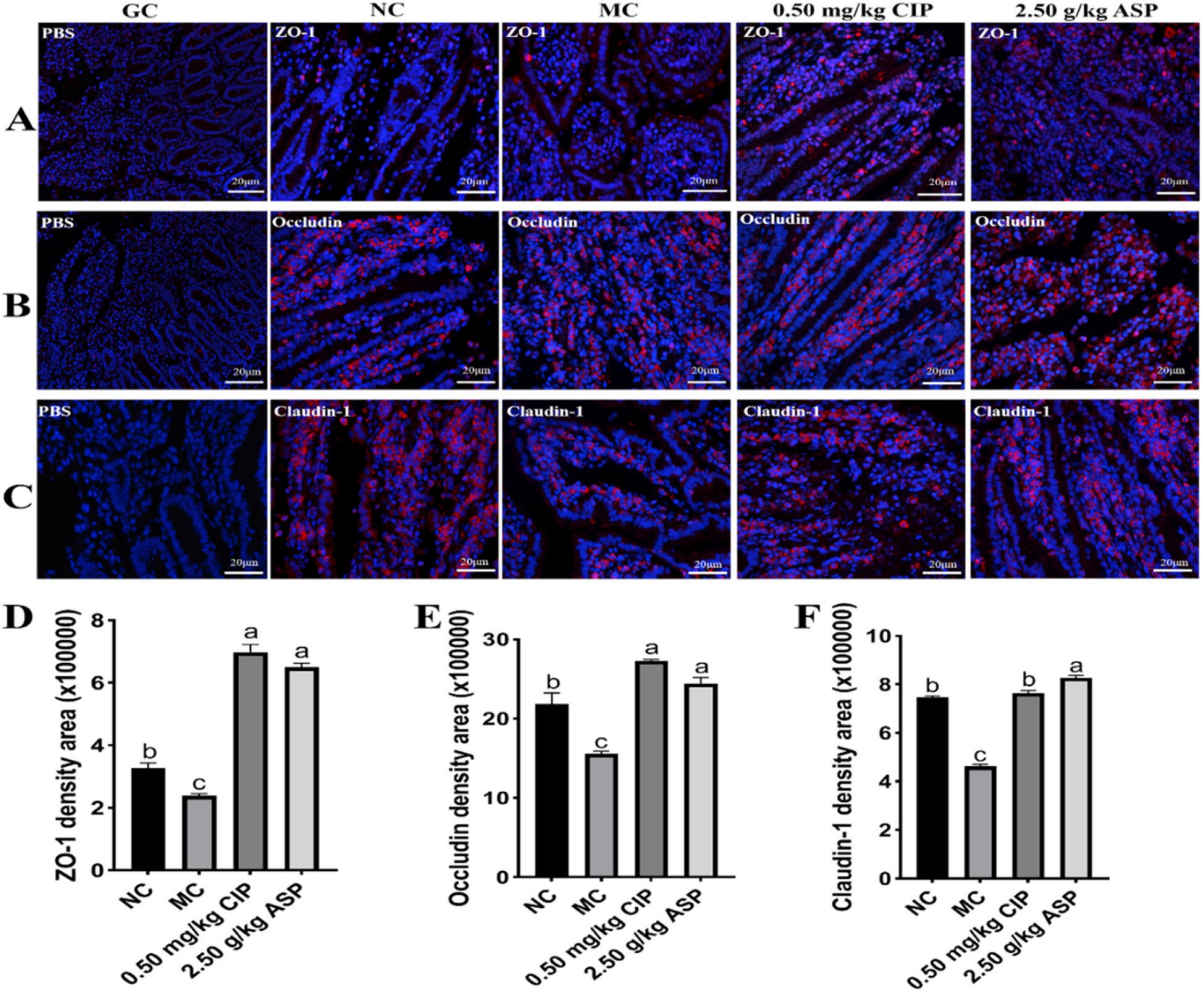
Intestinal tight junction proteins in pathogenic *E. coli* O_1_ induced diarrhea calf with ASP and CIP for 7 days (400 ×). Immunofluorescence staining of (**A**) ZO-1, (**B**) Occludin, and (**C**) Claudin-1 in the ileum. **D**, **E**, **F** Each field of ZO-1, Occludin, and Claudin-1 from every group was quantified by using ImageJ. Scale bar = 20 μm; red, ZO-1, Occludin, and Claudin-1; blue, 4,6-diamidino**-**2**-**phenylindole counterstaining of the nuclei. Control group was denoted as CG; NC, Normal control group; MC, *E. coli* group; CIP, Ciprofloxacin group; ASP, Arula-7 powder group. ZO-1, Occludin and Claudin-1

There was a significant difference in tight junction-related components among the 4 groups at the ending of day 7 (Fig. 5). Pathogenic *E. coli* O_1_ reduced the expression of ZO-1 (Fig. 5A), Occludin (Fig. 5B), and Claudin-1 (Fig. 5C) in the ileum. The expressions of ZO-1 (Fig. 5D), Occludin (Fig. 5E), and Claudin-1 (Fig. 5F) in the ASP and CIP groups increased compared with the MC group (*P* < 0.05). ASP and CIP administration restored ZO**-**1, Occludin, and Claudin-1 expression in the ileum. The distributions of ZO**-**1, Occludin, and Claudin-1 were also estimated by immunofluorescence staining. Compared with the MC group, which showed clear and uniform positive staining of the ZO-1 (Fig. 5D), Occludin (Fig. 5E), and Claudin-1 (Fig. 5F) in the intestinal epithelium if compared with MC and NC groups at 7-day (*P* < 0.05), the MC group showed a decrease staining of both ZO-1, Occludin, and Claudin-1 in the ileum if compared with NC group (*P* < 0.05).

### *Effect of ASP administration improved intestinal CD4*^+^*T lymphocytes and their representative cytokines*

**Fig. 6:**
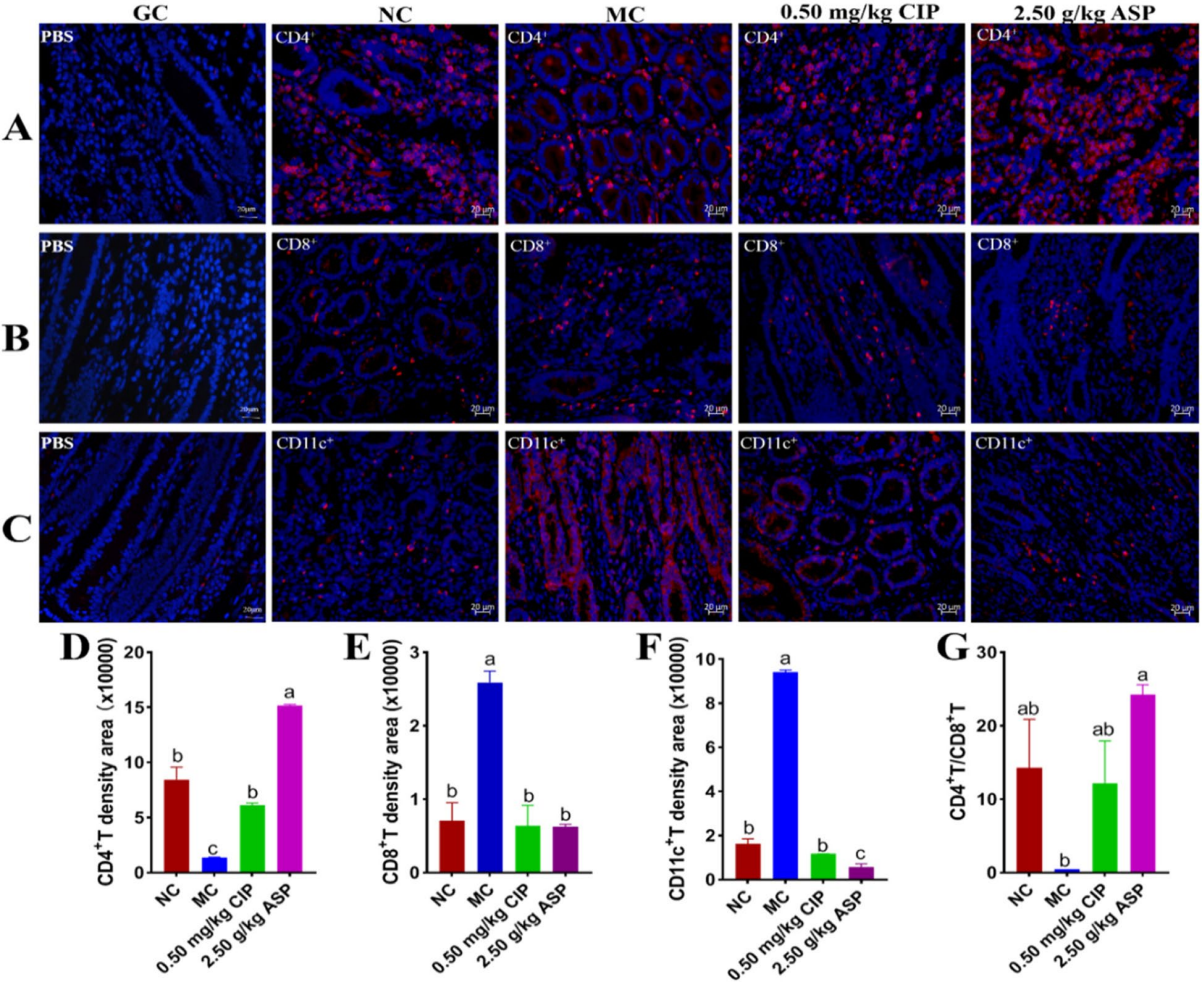
Intestinal lymphocyte surface molecular proteins in pathogenic *E. coli* O_1_ induced diarrhea calf with ASP and CIP for 7 days (400 ×). Immunofluorescence staining of **A** CD4^+^, **B** CD8^+^, and **C** CD11c^+^ in the ileum. **D**,** E**,** F** Each field of CD4^+^, CD8^+^, and CD11c^+^ from every group was quantified by using Image J. **G** The ratio of CD4^+^/CD8^+^T was significantly high in ASP group. Scale bar = 20 μm; red, CD4^+^, CD8^+^, and CD11c^+^; blue, 4,6**-**diamidino**-**2**-**phenylindole counterstaining of the nuclei. Control group was denoted as CG; NC, Normal control group; MC, *E. coli* group; CIP, Ciprofloxacin group; ASP, Arula-7 powder group

The distributions of CD4^+^T, CD8^+^T and CD11c^+^T were also estimated by immunofluorescence staining (Fig. 6). Oral *E. coli* O_1_ significantly reduced the expression of CD4^+^T in ileum (Fig. 6A), while the expression of CD8^+^T (Fig. 6B) and CD11c^+^T (Fig. 6C) significantly increased (*P* < 0.05). Compared with NC calves, the MC calves showed significantly more CD8^+^T (*P* < 0.05; Fig. 6E) and CD11c^+^T (*P* < 0.05; Fig. 6F) deposition in the ileum epithelium, whereas a dramatic reduction in the ileum accumulation was noted with ASP and CIP administration. Compared with the MC group, ASP group significantly increased CD4^+^/CD8^+^T (*P* < 0.05; Fig. 6G) and increased CD4^+^T (*P* < 0.05; Fig. 6D). In addition, ASP significantly increased the CD4^+^T and decreased CD11c^+^T (*P* < 0.05) but had no effect on CD8^+^T (*P* > 0.05) compared with that in NC group (*P* < 0.05). The CD4^+^T in the ASP group was significantly higher than those in the CIP group (*P* < 0.05). Our results showed that ASP and CIP administration reversed CD4^+^T expression in the ileum.

### ASP improved calf immune responses

**Fig. 7:**
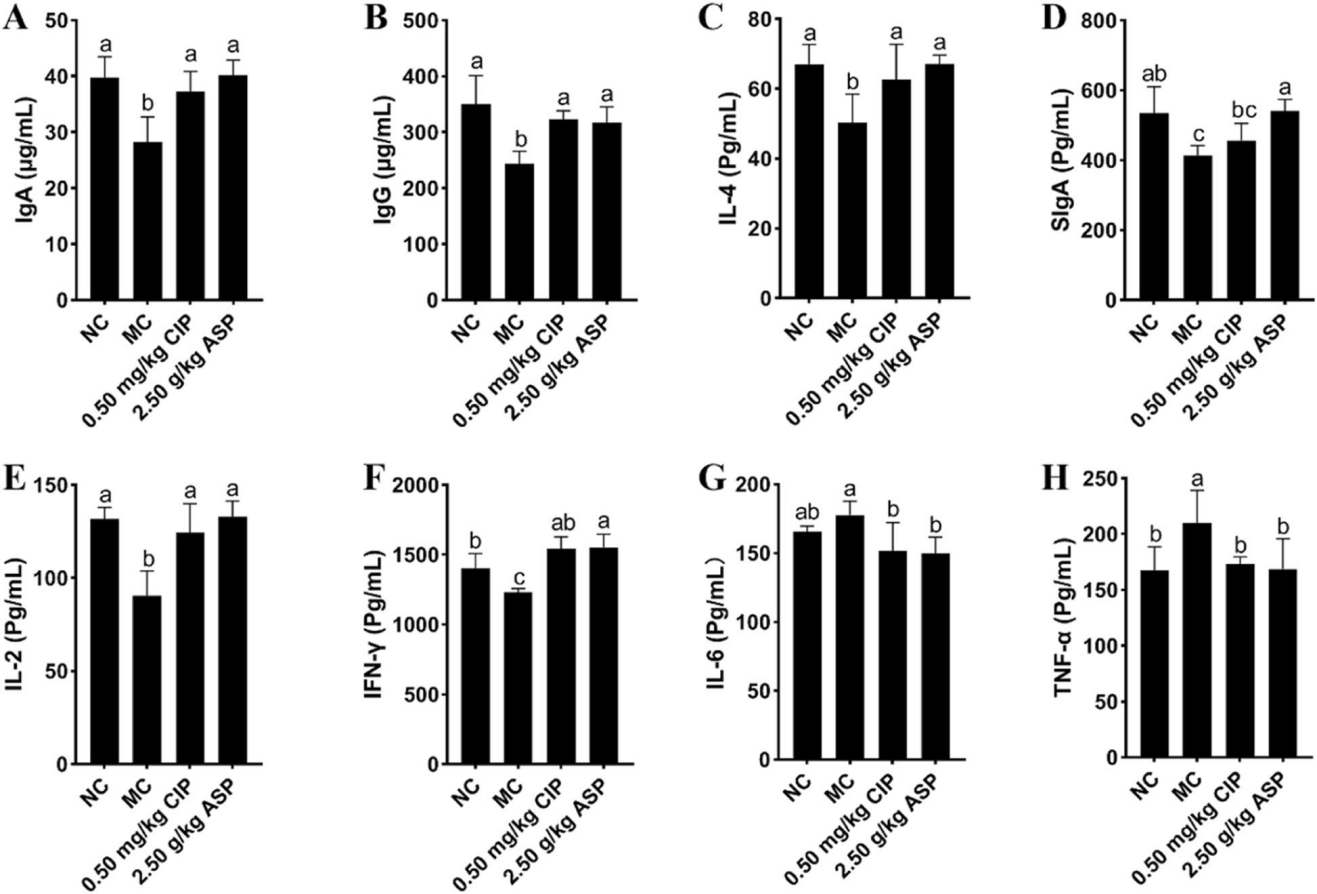
Effects of ASP treatment on calf immune markers. The levels of **A** serum IgA, **B** serum IgG, **C** serum SIgA, **D** serum IL-4, **E** serum IL-2, **F** serum IFN-γ, **G** serum IL-6, **H** serum TNF-α

Firstly, we investigated the effects of the ASP treatment on animal immunity, the serum IgA, IgG, SIgA, IL-4, IL-2, IFN-γ, IL-6, and TNF-α, were monitored (Fig. 7). ASP and CIP significantly increased IgA (Fig. 7A), IgG (Fig. 7B), IL-4 (Fig. 7D), IL-2 (Fig. 7E), and IFN-γ (Fig. 7F), and decreased IL-6 and TNF-α compared with that in MC group (*P* < 0.05). For the MC group, significantly reduced the levels of serum IL-6 (Fig. 7G) and TNF-α (Fig. 7H) comparing with NC group.

## Discussion

Intestinal development is a critical stage of growth for infants and newborn calves, and infection with pathogenic *E. coli* during intestinal development can cause intestinal barrier dysfunction, gut microbiota disruption, and inflammation, accompanied by a decline in the absorption capacity of the small intestine, and eventually leading to an increase in the incidence of diarrhea [7]. Pathogenic *E. coli* infection is associated with impairment of cellular immunity and an increased prevalence of gastrointestinal infection in newborn calves [2]. Therefore, the appropriate addition of plant-based drugs in the early stage seems to be crucial for enhancing the gut health of animals. Gan et al. reported that some plant extracts (e.g., curcumin) were highly effective in protecting the intestinal barrier function of mice [17]. In this work, we found that ASP powder as a natural antibacterial, anti-inflammatory, and immune enhancer, improved calf growth performance, reduced diarrhea, strengthened immunity, ameliorated intestinal barrier dysfunction and inflammation, and blocked the discharge of pathogenic microbes.

First, the calf was supplemented with Arula**-**7 powder in breast milk for 5 days after birth, then the calf was orally infected with pathogenic *E. coli* O_1_ and the drug was administered for another 2 days. After the 7-day trial, the incidence of diarrhea in the ASP group was low, which may be related to decreased colonization of pathogenic *E. coli* O1 [6]. Compared with the MC group, ASP increased the ADG, indicating that the weight was improved. In addition, ASP significantly reduced the rate of diarrhea in calves infected with pathogenic *E. coli* O_1_. Compared with the NC group, in the MC group, the ADG and final weight were reduced due to the unbalanced intestinal flora. In the current study, the weight gain, immunity, and diarrhea rate of animals were related to intestinal mucosa permeability, intestinal morphology, and intestinal flora balance [33]. Further analysis showed that milk supplementation with ASP significantly increased the contents of TP, ALB, and Glu in the serum, and reduced the content of BUN. These findings suggest that ASP supplementation affected the growth and production performance of calves. The effect of ASP on antioxidation indices is consistent with a prior study of diarrhea in rabbits [34].

In addition, the structure of the epithelial barrier was impaired, and the tight junctions of the intestinal epithelium were destroyed, thereby destroying the polarity of the intestinal epithelial cells and resulting in the substance spilling into the epithelial cell gap [35]. Protecting the ZO**-**1 protein in the intestine plays an important role in maintaining the tight junctions of intestinal epithelial cells [36]. Occludin, a transmembrane protein, is positively correlated with the structural integrity of the intestinal epithelium and intestinal epithelial barrier function [37]. After EPEC infection of T84 human intestinal epithelial cells, the close relationship between claudin-1, occludin and ZO**-**1 gradually decreased, their positions gradually changed, and the structure was damaged [38]. Huangqin decoction has been reported to significantly inhibit colitis and improve tissue damage [5]. In this study, ASP powder improved the intestinal morphology of calves infected with pathogenic *E. coli* O_1_ and increased the expression of the tight junction proteins ZO-1, Occludin, and Claudin-1. Currently, there is no relevant research on the effect of ASP powder on the intestinal morphology of calves infected with pathogenic *E. coli* O_1_. Therefore, this study may be the first to report that ASP powder can significantly improve the intestinal tight junctions by increasing the expression of ZO-1, Occludin, and Claudin-1 in the intestine morphology. The ability of ASP powder to improve calf diarrhea by pathogenic *E. coli* O_1_ may be due to its effects of improving intestinal morphology and increasing tight junction proteins.

The intestine plays an important role in protecting the animal's body from bacteria and acting as an immune barrier [39]. To investigate ASP-induced immunity regulation in calves, our work analyzed changes in several T cell populations in the intestinal tissues, CD4^+^T, CD8^+^T, CD11c^+^T, and several serum cytokines, IFN-γ, IL-4, IL-6, IL**-**2, TNF-α, and IL-6, as well as serum IgG, IgA, and SIgA. An increase in CD11c^+^T and CD8^+^T cells on the intestinal mucosa has already been proven in animal models of virus infection [40]. The various functions exhibited by CD8 + T are primarily determined by changes in the microenvironment induced by inflammation [18]. Although high expression of CD8 + T reduced IFN-γ levels, its mechanism was likely to occur in a cell–cell contact-dependent fashion [41]. Compared with those in the MC group, the levels of 3 cytokines serum IL-2, IL-4 and IFN-γ, and serum IgG, IgA, and SIgA, as well as tissue CD4^+^T of the ASP group had increased (*p* < 0.05). Thus, the increased CD8^+^T, CD11c^+^T cell populations, and the increased expression of IL-6 and TNF-α observed in the tissue of the MC group in the present study might be an indication of restricted immunity between the intestinal lumen and epithelium as a physiological response to diarrhea, but ASP opposes diarrhea. In the encephalitis models, the CD8^+^T inhibit the expansion of CD4^+^T cells via cell–cell contact [42]. Here, we observed that the concentration of CD4 + T in the intestinal tissue of ASP-treated calves was significantly higher than that in the NC and MC groups. Immunoglobulin (Ig) has long been recognized as the first immune barrier against the invasion of pathogenic microorganisms at the mucosa [43]. It can prevent pathogenic microorganisms from adhering to the surface of intestinal epithelial cells and neutralize bacterial toxins [44]. IgG binds to gram-negative bacteria in the intestinal lumen to reduce the risks of bacterial translocation, intestinal damage and systemic infection [45]. Additionally, the levels of IL-6 and TNF-α were significantly reduced in the ASP and CIP groups, and SIgA was significantly higher in the ASP group than in the CIP group. Moreover, although the levels of intestinal tissue CD4^+^T and serum IL-2, IL-4, and IFN-γ, and serum IgG, IgA, and SIgA in the MC group were lower than those in the NC group, their levels increased after ASP and CIP treatment, indicating that ASP and CIP could strengthen the immunity of calves. However, opposite observations were reported by Liu et al. [46].

Recent studies have shown that herbal medicines can directly lead to intestinal microflora shift and reduce inflammation, thereby improving the gut health of animals [15]. The gut microbiome has long been considered to be linked to immune system function and regulation [34]. The reduction of *Bacteroides* in the gut of obese humans and animals reduces the abundance of *Firmicutes*, suggesting that these major phyla may play a role in obesity-related inflammation [47]. A lower ratio of *Firmicutes* to *Bacteroidetes* is a sign of intestinal health. Compared with the NC group, the *Firmicutes* to *Bacteroidetes* ratio in the cecal contents of calves in the MC group was increased, which is consistent with the high diarrhea rate in the MC group. We observed that ASP increased the abundance of *Bacteroides* and *Firmicutes* in the cecal contents of calves infected with pathogenic *E. coli* O_1_, reduced the abundance of *Firmicutes* and *Proteobacteria* in the cecal and colonic contents, and increased the abundance of *Bacteroides* in colonic contents. Intriguingly, any alteration in the physical or chemical colonic contents may affect the resident bacterial structure and potentially promote or inhibit the establishment of pathogenic bacteria [34]. In this study, ASP increased the relative abundance of the genera *Faecalibacterium*, *Alloprevotella*, and *Bacteroides* in the cecal and colonic contents and increased the relative abundance of the order *Bacteroidales*, while decreasing the abundance of *Proteobacteria*, *Selenomonadales*, *Enterobacteriales*, and *Clostridiales* in the cecal and colonic contents. The genera *Faecalibacterium* and *Alloprevotella* produce most of the amino acids that calves obtained through their intestines. These strains promote weight gain and reduce intestinal inflammation and diarrhea [48]. In addition, *Faecalibacterium* promotes the differentiation of T and B lymphocytes into plasma cells and increases the metabolism of T and B cells, thereby enhancing the production of host antibodies [49]. Therefore, ASP could improve the immune function of calves infected with pathogenic *E. coli* O_1_ by increasing the relative abundance of *Faecalibacterium*. In addition, ASP may reduce the incidence of diarrhea caused by *E. coli* O_1_ by decreasing the abundance of *Proteobacteria*, *Selenomonadales*, and *Enterobacteriales*.

In the diarrhea of calves, a close link between intestinal flora and feces flora was observed, and the changes in the flora associated with diarrheal disease were similar between the two species, characterized by a decrease in *Proteobacteria* and *Bacteroidetes*. In the current study, ASP increased the relative abundance of *Fusobacteria* and *Lactobacillus* in the rectal feces of calves. *Lactobacillus*, a probiotic, promotes weight gain; therefore, ASP promotes the weight gain of calves by altering the rectal microbiota. In addition, a previous study showed that *Lactobacillus* is involved in regulating the immune system, preventing diarrhea, and improving animal health [50]. On the one hand, it is plausible that ASP could improve the immune function of calves infected with pathogenic *E. coli* O_1_ by increasing the relative abundance of *Faecalibacterium*. On the other hand, ASP may reduce the incidence of diarrhea caused by *E. coli* by decreasing the number of *E. coli*. Therefore, the restoration of intestinal ecosystem function can potentially be used as an effective treatment strategy for calves infected with pathogenic *E. coli* O_1_. This may be because ASP can reducing the occurrence of diarrhea by reducing the abundance of *Proteobacteria* and increasing the abundance of *Faecalibacterium*. This further illustrates that ASP completely blocks the release of pathogenic microorganisms in the calf intestine, protects the environment, and avoids infection of other animals.

## Conclusions

Overall, the results of this study suggest that milk supplementation with ASP powder promoted weight gain, ameliorated diarrhea and inflammation, and improved immunity in calves infected with pathogenic *E. coli* O_1_ in a 7-day trial. These results provide a basis for the further development of green products that maintain the functional integrity of the intestinal mechanical and biological barriers in calves infected with pathogenic *E. coli* O_1_. These results provide a theoretical basis and experimental evidence for understanding the biological function of ASP powder and its potential for use in place of antibiotics.

## Data Availability

Raw 16S rRNA gene sequences for all samples used in this study have been deposited in the Sequence Read Archive (SRA) under project accession no. SUB6318504, SUB6321029, and SUB6321118.
